# Plasma MicroRNAs (miR-146a, miR-103a, and miR-155) as Potential Biomarkers for Rheumatoid Arthritis (RA) and Disease Activity in Iranian Patients

**DOI:** 10.31138/mjr.32.4.324

**Published:** 2021-12-27

**Authors:** Zahra Bagheri-Hosseinabadi, Mohammad Reza Mirzaei, Mohammad Reza Hajizadeh, Fateme Asadi, Mehdi Rezaeian, Mitra Abbasifard

**Affiliations:** 1 Molecular Medicine Research Center, Research Institute of Basic Medical Sciences, Rafsanjan University of Medical Sciences, Rafsanjan, Iran,; 2 Department of clinical Biochemistry, School of medicine, Rafsanjan University of Medical Sciences, Rafsanjan, Iran,; 3 Internal Medicine Resident, Department of Internal Medicine, Ali-Ibn Abi-Talib Hospital, Rafsanjan University of Medical Sciences, Rafsanjan, Iran,; 4 Department of internal Medicine, School of medicine, Ali-Ibn-Abi-Talib Hospital, Rafsanjan University of Medical Sciences, Rafsanjan, Iran

**Keywords:** rheumatoid arthritis, microRNA, whole blood sample, disease biomarker

## Abstract

**Background:**

Previous studies have shown that several microRNAs (miRNAs) are dysregulated in the whole blood as well as diverse cells and tissues from rheumatoid arthritis (RA) patients. The aim of the current study was to determine if the expression of miR-146a, miR-103a, and miR-155 in whole blood of RA patients could confer potential markers in evaluating of activity-severity of the disease in RA patients with established disease.

**Methods:**

Whole blood samples were obtained from 30 RA patients and 30 healthy subjects. The RNA content of blood samples was isolated, cDNA was synthesized, and transcript levels of miR-146a, miR-103a, and miR-155 were determined using Real-time PCR. The clinicopathological characteristics of the patients were also evaluated.

**Results:**

It was detected that expression level of miR-146a (fold change=1.85, *P*=0.004), miR-103a (fold change=2.44, *P*=0.0018), and miR-155 (fold change=1.94, *P*=0.0025) were significantly upregulated in the whole blood samples of RA patients in comparison to that of healthy subjects. Expression level of miRNAs was correlated with clinicopathological characteristics of the patients, including Disease Activity Score 28 (DAS28), Simple Disease Activity Index (SDAI), 28Tender Joint Count (TJC-28), 28Swollen Joint Count (SJC-28), C-reactive protein (CRP), Rheumatoid factor (RF), and anti-cyclic citrullinated peptide (anti-CCP) antibodies.

**Conclusions:**

Upregulated levels of miR-146a, miR-103a, and miR-155 in the whole blood samples of RA patients could confer a potential marker of activity-severity of the disease in RA patients with established disease.

## INTRODUCTION

Rheumatoid arthritis (RA) is a debilitating autoimmune disorder that is primarily characterized by chronic joint inflammation erosive arthritis, and joint destruction.^1^ Subsequently, constant pathological alterations can create joint damage and deformities, deterioration of joint function, and also critical disabilities, changing the quality of life of the patients.^2^ The occurrence of RA differs from 0.3% to 1.0% generally, and it is more prevalent in women and in developed countries.^3^ Although the precise cause of RA is not completely known, it has been demonstrated that multiple genetic, environmental, and immunological factors are associated with the susceptibility to RA.^4–8^ Today, although the investigation into remedies to treat RA is continuing, there is no cure for this disorder.^9^ Regarding the variable and different clinical presentation of the disorder, its determination is originally based on the revised American College of Rheumatology/European League Against Rheumatism (ACR/EULAR) classification criteria which involve the evaluation of various clinical and serum factors as anti-cyclic citrullinated peptide (anti-CCP) antibodies. and rheumatoid factor (RF).^10^ However, anti-CCP and RF have polled sensitivity and raised the level of these biomarkers are also found in other autoimmune diseases.^11,12^ Hence, it is of great significance to finding the new biomarkers to control disease activity and therapeutic response in RA patients.^13^

MicroRNAs (miRNAs) are short non-coding RNAs and broadly distributed in a variety of organs and cells, operating a critical role in regulating gene expression by binding to the 3’ untranslated region (UTR) sequences of the target mRNAs and either begin their degeneration or repress their translation.^14,15^ It is understood that miRNAs participate in the event and development of many disorders, including cardiovascular diseases, autoimmune diseases, and cancers.^16,17^ In 2007, Bhanji and collaborators first found that miRNAs might play a function in RA progression.^18^ Then, following investigations divulged the dysregulation of certain miRNAs, including miR-146a and miR-155 within inflamed joints and in the peripheral circulation of RA patients.^19,20^ Recently, the roles of miR-146a, miR-103a, and miR-155 in RA have become a popular research topic, and levels of expression of these molecules have been investigated in synovial tissues, fibroblasts, and peripheral blood-derived mononuclear cells (PBMC) of RA patients.^21^ Nevertheless, there is a lack of reports about the association between the level of these miRNAs in plasma of RA patients and the activity of the disease. In this study, the expression of miR-146a, miR-103a, and miR-155 in plasma of RA patients was investigated to evaluate the roles of these miRNAs as potential markers in appearance, severity, and activity in RA patients with established disease.

## MATERIALS AND METHODS

### Study design

This observational case-control study was performed in the period from February to August 2020. The work was approved by the Ethics Committee from Rafsanjan University of Medical Sciences. This study was conducted according to the Declaration of Helsinki for investigations in humans. Before the procedure, informed approval was received from all participants.

### Patients and controls

The study was conducted on 30 RA patients attending the outpatient in the Rheumatology clinic of the Ali-Ebne-Abitaleb Hospital. The patients were diagnosed according to the American College of Rheumatology (ACR) classification criteria.^22^ Evaluation of RA patient’s disease activity was performed using Disease Activity Score 28 (DAS28). Clinical rheumatic data, including duration of disease, morning stiffness, number of tender swollen joints, extra-articular signs, and Simplified Disease Activity Index (SDAI) for Rheumatoid Arthritis score were documented for each subject. Patients with any other chronic debilitating diseases, chronic disorder, and pregnant subjects were excluded from the study. As the control group, 30 age- and sex-matched healthy individuals with no autoimmune disorders and familial history of autoimmunity, immunodeficiency, and malignancy were included.

### Laboratory investigations

#### Sample collection

Blood samples (5 ml) were collected in the EDTA-coated vials to separate plasma. A part of samples was used for analysis of laboratory parameters, including C-reactive protein (CRP), erythrocyte sedimentation rate (ESR), complement C3 and C4, rheumatoid factor (RF), and anti-cyclic citrullinated peptide (anti-CCP). Samples were centrifuged and stored at −20°C until analyses. The other part of plasma was frozen in tubes containing EDTA at −20°C until quantification of miRNA expression.

#### RNA isolation

RNA was isolated from blood using the blood MicroRNA extraction kit “miRNA Mini Kit” (Parsgenoum, Tehran, Iran) following the manufacturer’s guidance. Then, the purity of RNA was determined by agarose gel electrophoresis. The absorbance of samples was estimated by the UV-Vis spectrophotometer. Samples were stored at −20°C until further processed.

#### cDNA synthesis and quantitation of miRNAs by Real-time PCR

cDNA synthesis was conducted using miRNA amplification kit (Parsgenoum, Tehran, Iran) according to the manufacture’s protocol. cDNAs were manufactured in two steps, including the addition of a poly-adenine (poly-A) tail and next specific cDNA synthesis for all miRNAs. At the primary step, a proper volume of RNA was incubated with 2 μL of 10× buffer, 1 μL of adenosine triphosphate, 0.5 μL of the poly-A polymerase, and nuclease-free water up to 10 μL at 37°C for 10 minutes. Afterward, 5.5 μL of polyadenylated RNA was mixed with 2 μL of 5× buffer, 0.5 μL of reverse transcriptase, 1 μL of the linear primer, and 1 μL of deoxynucleotide triphosphate. The mixtures were incubated in a thermal cycler at 42°C for 60 minutes, followed by 85°C for 5 seconds. The miRNAs were quantified in duplicate reactions using a Rotor-gene Q thermal cycler (Qiagen, Hilden, Germany). Reactions were performed in final 20 μL volumes, including 10 μL of SYBR Green PCR Master Mix (TaKaRa, Kusatsu, Japan),1 μL of undiluted cDNA, 0.5 μL of forward primer, 0.5 μL of universal reverse primer, and 8 μL of nuclease-free water (CinnaGen, Tehran, Iran). The thermal profile of three-stepped qPCR was set at 95°C for 30 seconds as hold time, followed by 40 cycles of denaturation at 95°C for 5 seconds, annealing at 60°C for 20 seconds, and extension at 72°C for 30 seconds. Then melting curves were plotted at temperatures varying from 55 to 99°C. The expression of the U6snRNA was utilized as an endogenous control for data normalization.

### Statistical analysis

Statistical analyses of data were conducted using SPSS (Statistical Program for Social Science) version 22. Quantitative variables were presented as the mean ± standard deviation (SD) and qualitative variables were reported as numbers or percentages. The variation at baseline levels of miRNAs between patients and the control group was evaluated by applying the Mann-Whitney U test. Correlations with miRNA levels and clinical factors were examined by Spearman’s *rho* test. Statistical significance was established as a *P* value < 0.05.

## RESULTS

### Baseline data and laboratory findings

The demographic data and clinical presentations of the RA patients and healthy subjects are shown in **Table 1**. In the RA group, 19 (63.3%) females and 11 (36.7) males were included, while healthy control group was composed of 18 (60%) females and 12 (40%) males. The mean age of RA patients and healthy controls was 48.54±13.18 and 43.87±11.64 years, respectively. In the patients and control group, there were 13 (43.3%) and 13 (43.3%) smoker individuals. Disease duration of the RA patients was 11.5±5.8 years. Regarding the disease severity indexes, DAS28, SDAI, 28Tender Joint Count, and 28Swollen Joint Count were 3.41±1.24, 50.44±23.12, 3.01±2.14, and 2.74±2.04, respectively. The ESR and CRP levels of the RA patients were 20.44±15.46 mm/h and 5.23±2.44 mg/L, respectively. In the patient group, titters of RF and anti-CCP were 31.45±8.54 IU/ml and 36.12±12.64 IU/ml, respectively. Increased (abnormal) levels of C3 and C4 proteins of complement were detected in the 26 (86.6%) and 25 (83.3%) patients, respectively. Regarding the drug regimen of the patients, 21 (70%), 23 (76.6%), and 5 (16.6%) patients were using Corticosteroid, Methotrexate, and Biologics, respectively.

**Table 1.** Characteristics of rheumatoid arthritis patients.

**Characteristics**	**RA patients (n= 30)**	**Controls (n= 30)**
Gender, Female/Male	19 (63.3%)/11 (36.7)	18 (60%)/12 (40%)
Smoker/Non-smoker	13 (43.3%)/17 (56.7%)	13 (43.3%)/17 (56.7%)
Age, year (Mean±SD)	48.54±13.18	43.87±11.64
Disease duration years (Mean±SD)	11.5±5.8	-
DAS28	3.41±1.24	-
SDAI	50.44±23.12	-
28Tender Joint Count	3.01±2.14	-
28Swollen joint count	2.74±2.04	-
ESR (mm/h)	20.44±15.46	-
CRP (mg/L)	5.23±2.44	-
RF (IU/ml)	31.45±8.54	-
Anti-CCP (IU/ml)	36.12±12.64	-
C3 Complement, Increased levels	26 (86.6%)	-
C4 Complement, Increased levels	25 (83.3%)	-
Corticosteroid use	21 (70%)	-
Methotrexate use	23 (76.6%)	-
Biologics use	5 (16.6%)	-

RA: Rheumatoid arthritis; DAS28: Disease activity score; SDAI: Simplified disease activity index; ESR: Erythrocyte sedimentation rate; CRP: C-reactive protein; RF: Rheumatoid factor; Anti-CCP: Anti-cyclic citrullinated peptide; SD: Standard deviation

## miRNA EXPRESSION LEVEL

**Figure 1.**
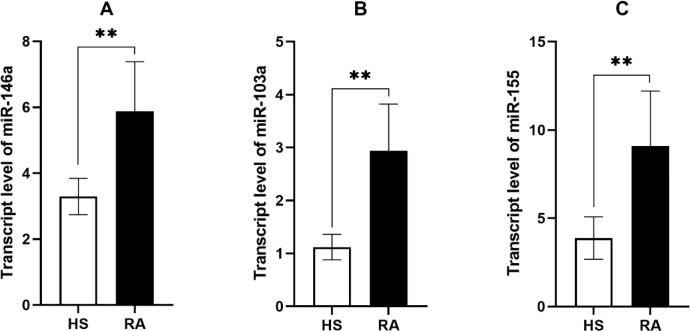
Expression levels of miRNAs. The whole blood samples were obtained from 30 RA patients and 30 healthy controls. Quantitative Real-time PCR was used to measure the expression levels of miR-146a (A), miR-103a (B), and miR-155 (C) in the samples (** shows *P*<0.01).

It was detected that expression level of miR-146a was upregulated significantly (fold change= 1.85, *P*= 0.004) in the whole blood samples of RA patients in comparison to that of healthy subjects (**Figure 1A**). Additionally, the transcript level of miR-103a indicated significant overexpression (fold change= 2.44, *P*= 0.0018) in the hole blood samples of RA cases compared with that of healthy controls (**Figure 1B**). Similarly, the transcript level of miR-155 had significantly upregulated (fold change= 1.94, *P*= 0.0025) amounts in the hole blood samples of RA group compared with that of healthy control group (**Figure 1C**).

The comparison of the expression level of miRNAs in RA patients based on gender, smoking, complement C3 and C4 levels, and medication indicated no statistically significant differences (**Table 2**).

**Table 2.** Comparison of miRNA expression levels in RA patients with and without specific characteristics.

**Characteristics**	**miR-146a fold change (*P* value)**	**miR-103a fold change (*P* value)**	**miR-155 fold change (*P* value)**
Female *vs*. Male	1.24 (0.272)	1.16 (0.348)	1.02 (0.877)
Smoker *vs*. Non-smoker	0.98 (0.544)	0.87 (0.155)	0.90 (0.233)
Increased C3 *vs*. Normal C3	0.94 (0.349)	0.91 (0.248)	0.88 (0.655)
Increased C4 *vs*. Normal C4	0.90 (0.460)	0.97 (0.714)	0.85 (0.198)
Corticosteroid user *vs*. Corticosteroid non-user	1.05 (0.544)	1.10 (0.404)	1.14 (0.277)
Methotrexate user *vs*. Methotrexate non-user	1.08 (0.280)	1.11 (0.127)	1.09 (0.204)
Biologics user *vs*. Biologics non-user use	1.02 (0.323)	1.15 (0.216)	1.25 (0.147)

DAS28: Disease activity score; SDAI: Simplified disease activity index; ESR: Erythrocyte sedimentation rate; CRP: C-reactive protein; RF: Rheumatoid factor; Anti-CCP: Anti-cyclic citrullinated peptide

### Correlation analysis

It was observed that expression levels of miR-146a (rho= 0.51, *P*= 0.014), miR-103a (rho= 0.66, *P*= 0.010), and miR-155 (rho= 0.59, *P*= 0.001) had significantly positive correlation with DAS28 of the RA patients. Furthermore, a significantly direct correlation was found between expression levels of miR-146a (rho= 0.61, *P*= 0.021), miR-103a (rho= 0.50, *P*= 0.035), and miR-155 (rho= 0.58, *P*= 0.041) and SDAI in RA subjects. In addition, both 28Tender Joint Count and 28Swollen Joint Count were positively correlated with the expression levels of miR-146a, miR-103a, and miR-155 in RA patients. Although ESR levels had non-significant correlation with expression levels of the miRNAs, CRP level had significantly positive correlation with expression level of miR-146a (rho= 0.41, *P*= 0.042). RF titre had significantly positive correlation with expression levels of miR-146a (rho= 0.49, *P*= 0.015) and miR-103a (rho= 0.41, *P*= 0.021). Anti-CCP titre showed significantly positive correlation (rho= 0.55, *P*= 0.019) with expression level of only miR-146a (**Table 3**).

**Table 3.** Correlation analysis of the miRNA expression levels and clinicopathological properties of the RA patients.

**Characteristics**	**miR-146a *rho* (*P* value)**	**miR-103a *rho* (*P* value)**	**miR-155 *rho* (*P* value)**
Age	0.24 (0.233)	0.31 (0.214)	0.35 (0.388)
Disease duration	0.12 (0.511)	0.24 (0.087)	0.20 (0.281)
DAS28	0.51 (0.014)	0.66 (0.010)	0.59 (0.001)
SDAI	0.61 (0.021)	0.50 (0.035)	0.58 (0.041)
28Tender Joint Count	0.48 (0.047)	0.55 (0.030)	0.63 (0.003)
28Swollen Joint Count	0.49 (0.014)	0.53 (0.031)	0.54 (0.011)
ESR (mm/h)	0.31 (0.087)	0.38 (0.066)	0.19 (0.188)
CRP (mg/L)	0.41 (0.042)	0.31 (0.088)	0.33 (0.163)
RF (IU/ml)	0.49 (0.015)	0.41 (0.021)	0.35 (0.221)
Anti-CCP (IU/ml)	0.55 (0.019)	0.31 (0.078)	0.27 (0.055)

DAS28: Disease activity score; SDAI: Simplified disease activity index; ESR: Erythrocyte sedimentation rate; CRP: C-reactive protein; RF: Rheumatoid factor; Anti-CCP: Anti-cyclic citrullinated peptide

## DISCUSSION

The aim of the current study was to determine if the expression of miR-146a, miR-103a, and miR-155 in whole blood of RA patients could be potential markers of activity-severity of the disease in RA patients with established disease. Our experiments revealed that the transcript levels of all three miRNAs had upregulation in the whole blood samples of the RA patients compared to the controls. Moreover, the expression level of miRNAs was correlated with clinicopathological characteristics of the patients, including DAS28, SDAI, 28Tender Joint Count, 28Swollen Joint Count, CRP, RF, and Anti-CCP.

Inflammatory cytokines, such as interleukin (IL)-1, IL-17, and tumour necrosis factor (TNF)-α have been reported to modulate the expression of several miRNAs, like miR-146a and miR-155 in different cells from RA patients, including T cells, peripheral blood mononuclear cells (PBMCs), synovial fibroblasts, and synovial tissues.^23^ On the other hand, these miRNAs are able to control the signalling pathways of inflammatory, leading to modulation of the bone homeostasis as well as cell differentiation in the synovial region.^23–25^ As a result, miRNAs are critically involved in the controlling of inflammation, growth of synoviocytes, and osteoclastogenesis, hence impressing the disease activity and severity in RA patients.^24–26^ Thus, miRNAs may confer an important epigenetic regulatory factor in the modulation of immune tolerance as well as initiation and perpetuation of the RA disease.

Zhou et al. revealed that diminished upregulation of miR-146a and miR-155 in response to T cell stimulation was observed in the regulatory T (Treg) cells from RA patients. Diminution of miR-146a expression was detected specially in patients with active disease and had correlation with inflammation in the joints. In the active RA patients, decreased expression of miR-146a (targeting the signal transducer and activator transcription 1 [STAT1]) resulted in development of a pro-inflammatory phenotype in Treg cells.^27^ Hence, miR-146a in RA patients promotes a pro-inflammatory phenotype of Treg cells and contributes to the pathogenesis of RA. miR-155 is a critical regulator of immune cells and plays critical roles in the pathogenesis of RA. Studies have demonstrated that expression of miR-155, which targets the transcription factor suppressor of cytokine signalling (SOCS) 1 and led to upregulation of inflammatory cytokines, was increased in RA patients and animal models of arthritis.^28^

Even though abnormal expression profile of miRNA has been widely investigated in the PBMCs and peripheral blood of RA patients,^25,29^ little has determined the potential of the miRNAs in determining the diseases progression in these patients. One of the main advantages of utilising whole blood samples to measure miRNA transcript levels is that this method maintains the rich compositional makeup of the blood, hence conferring the highest unbiased exhibition of this tissue. Despite that application of whole blood samples may be suitable for biomarker identification, its main disadvantage is that it is not possible to determine the corresponding cellular source of released miRNA. Considering the heterogeneity of immune cells in the blood circulation, the definition of mechanistic hypotheses is difficult. In order to resolve this difficulty, a bulk of the past investigations on the whole blood miRNA profiling in RA has also concentrated on the PBMCs as well as other immune cells.^25^ Nonetheless, researches trying to identify the correlation of miRNA expression in the PBMC and whole blood samples have generated incongruous finding.^30^ Atarod et al. revealed that the expression profiling of miR-146a and miR-155 in the whole blood sample was not in harmony with that of PBMCs.^31^ The RNA isolation techniques, the count of blood cells, and haemolysis of red blood cells might be the source of conflicting results.^32^

Previous studies on miRNA expression in RA have concentrated on differences in miR-146a and miR-155 expression, which both of them were shown to be upregulated in the PBMCs and synovial tissues from RA patients (25, 30, 33). Anaparti et al. evaluated the expression of miRNAs in whole blood as well as PBMCs, and observed that the levels of miR-103a-3p, miR-155, and miR-146a-5p were increased in both samples in RA patients as well as in the anticitrullinated protein antibodies (ACPA)-positive asymptomatic first-degree relatives (FDRs) without clinical presentations of arthritis.^34^ Our results also indicated that expression levels of miR-103a, miR-155, and miR-146a were higher in the whole blood of RA patients compared with healthy controls. Interestingly, the expression level of miRNAs had correlation with clinicopathological manifestations of the patients, including DAS28, SDAI, 28Tender Joint Count, 28Swollen Joint Count, CRP, RF, and Anti-CCP. As a consequence, these miRNAs might confer a potential to be used as markers for determining the severity of the disease.

## CONCLUSIONS

Considering all the facts, here we intended to determine if the transcript levels of miR-146a, miR-103a, and miR-155 in the whole blood samples of RA patients could serve as potential markers in evaluating the activity-severity of the RA disease. It was revealed that the transcript levels of all three miRNAs was overexpressed in the whole blood samples of the RA patients compared to the healthy controls. Additionally, the expression level of miRNAs had correlation with clinicopathological characteristics of the patients, including DAS28, SDAI, 28Tender Joint Count, 28Swollen Joint Count, CRP, RF, and Anti-CCP. That notwithstanding, further evaluations might be helpful in discovering a valid biomarker for the RA disease.
